# Novel Oral Polio Vaccine Type 2 Use for Polio Outbreak Response: A Global Effort for a Global Health Emergency

**DOI:** 10.3390/pathogens13040273

**Published:** 2024-03-23

**Authors:** Feyrouz Damji Kurji, Ananda Sankar Bandyopadhyay, Simona Zipursky, Laura V. Cooper, Chris Gast, Margaret Toher, Ralf Clemens, Sue Ann Costa Clemens, Rayasam Prasad, Adriansjah Azhari

**Affiliations:** 1 FDK Consulting, LLC, Watford WD17 1HP, UK; feyrouz@fdkconsult.com; 2 Bill & Melinda Gates Foundation, Seattle, WA 98109, USA; simona.zipursky@gatesfoundation.org (S.Z.); ray.prasad@gatesfoundation.org (R.P.); 3 MRC Centre for Global Infectious Disease Analysis, School of Public Health, Imperial College London, London SW7 2BX, UK; l.cooper@imperial.ac.uk; 4 Center for Vaccine Innovation and Access, PATH, Seattle, WA 98121, USA; cgast@path.org (C.G.); mtoher@path.org (M.T.); 5 International Vaccine Institute IVI, Seoul 08826, Republic of Korea; 6 Institute for Global Health, Siena University, 53100 Siena, Italy; sueannclemens@outlook.com; 7 PT Bio Farma, Kota Bandung 40161, Indonesia; adriansjah@biofarma.co.id

**Keywords:** poliovirus, novel oral poliovirus vaccine, emergency use listing, WHO prequalification, vaccine, public health emergency of international concern

## Abstract

A sharp rise in circulating vaccine-derived poliovirus type 2 (cVDPV2) outbreaks in the years following the cessation of routine use of poliovirus type 2-containing oral polio vaccine and the trend of seeding new emergences with suboptimal vaccination response during the same time-period led to the accelerated development of the novel oral polio vaccine type 2 (nOPV2), a vaccine with enhanced genetic stability and lower likelihood of reversion to neuroparalytic variants compared to its Sabin counterpart. In November 2020, nOPV2 became the first vaccine to be granted an Emergency Use Listing (EUL) by the World Health Organization (WHO) Prequalification Team (PQT), allowing close to a billion doses to be used by countries within three years after its first rollout and leading to full licensure and WHO prequalification (PQ) in December 2023. The nOPV2 development process exemplifies how scientific advances and innovative tools can be applied to combat global health emergencies in an urgent and adaptive way, building on a collaborative effort among scientific, regulatory and implementation partners and policymakers across the globe.

## 1. Introduction

The novel oral polio vaccine type 2 (nOPV2) was the first vaccine to be granted an Emergency Use Listing (EUL) by the World Health Organization (WHO) Prequalification Team (PQT) in November 2020, leading eventually to full licensure by the Indonesian regulatory authority (Badan POM) and WHO prequalification (PQ) in December 2023 [1]. The first successful use of a unique clinical development and regulatory pathway and the subsequent massive rollout of nOPV2 have had a meaningful impact on the global polio eradication effort: use of the vaccine under the EUL from March 2021 to December 2023 allowed for the administration of approximately one billion doses of nOPV2 across 35 countries [1]. Throughout this period, the enhanced genetic stability of nOPV2 was demonstrated, a key feature of its design, compared with Sabin oral polio vaccine (OPV).

OPVs induce primary intestinal mucosal immunity to reduce replication of polioviruses in the gut when subsequently exposed to the virus, making their use in outbreak scenarios an important tool in halting poliovirus transmission. However, in rare circumstances, OPV strains can revert to virulence, causing paralysis in vaccine recipients and close contacts, a phenomenon known as vaccine-associated paralytic poliomyelitis (VAPP). Moreover, OPVs can revert following replication in the human gut and lose key attenuating mutations in the process, leading to the generation of vaccine-derived polioviruses (VDPVs). In areas with persistently low vaccination coverage, such revertant OPV strains can propagate from person to person and generate circulating vaccine-derived polioviruses (cVDPVs), which have regained the transmissibility properties of wild polioviruses and thus can cause outbreaks of paralytic disease. Outbreaks from wild polioviruses and cVDPVs are indistinguishable. The determining factors of the scale and impact of polio outbreaks are affected by differences in the underlying factors in the sub-populations where outbreaks occur, such as background immunity, population density, and the status of sanitation and hygiene. In addition, based on the primary serotype associated with the polio outbreak, the proportion of paralytic cases might vary, with type 1 being the most paralytic strain and type 2 the least [2]. Wild type 2 and wild type 3 polioviruses were certified to be eradicated in 2015 and 2019, respectively, once indigenous transmission of these viruses had been interrupted globally, according to criteria followed by the global certification committee for polio eradication.

The strategy of responding to cVDPV type 2 (cVDPV2)—the leading cause of cVDPV outbreaks globally—with Sabin OPV type 2 (OPV2), in monovalent (mOPV2) or trivalent (tOPV) formulations, has been largely successful in stopping transmission; however, due to the inherent genetic instability of Sabin strains and waning population immunity following the global cessation of the routine use of OPV2 since April 2016, an increasing number of new cVDPV2 outbreaks are attributable to OPV2 use [3]. While inactivated poliovirus vaccine (IPV) used in routine immunization and occasionally in outbreak response protects against paralytic disease from type 2 polioviruses, it provides limited primary intestinal mucosal immunity necessary to stop outbreaks of cVDPV2 [4,5]. Therefore, the idea of an OPV with enhanced genetic stability at key primary attenuation sites and with lower risk of reversion to paralytic and outbreak-inducing forms has long been considered a potential game changer for achieving and sustaining eradication of all forms of polioviruses.

## 2. nOPV2 Development

Early work for nOPV2 development began in 2011 with a scientific consortium convened by the Bill & Melinda Gates Foundation (BMGF) to address the risk of cVDPV and VAPP associated with the use of Sabin OPVs. The primary objective of the consortium was to develop a genetically modified vaccine that is as effective immunologically and as affordable as the Sabin OPV but has lower risk of reversion to neurovirulent forms, with an aim to minimize the risk of VAPP and to limit the rate of seeding of new outbreaks of cVDPV. Researchers from the National Institute for Biological Standards and Controls (NIBSC) in the United Kingdom, the US Centers for Disease Control and Prevention (US CDC), the US Food and Drug Administration (US FDA), and the University of California at San Francisco (UCSF) were the primary constituents of the consortium that collaborated to design, produce, and test several novel OPV strains in a variety of pre-clinical studies.

**Figure 1:**
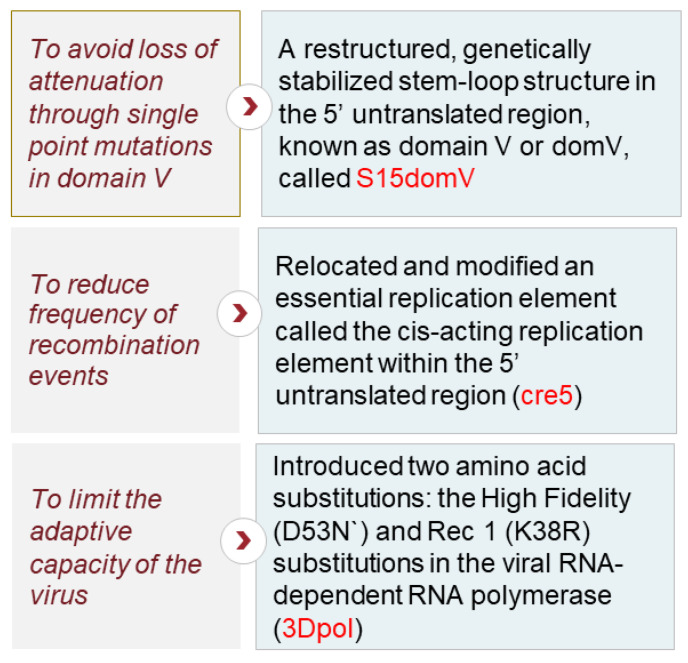
Modifications made to the Sabin Oral Polio Vaccine Type 2 virus genome to design novel Oral Polio Vaccine Type 2.

To increase the genetic stability of the vaccine, modifications were made to targeted sites on the vaccine strain’s genome. Some of the final, key modifications are listed in Figure 1, depicting the rationale behind the changes in selected nOPV2 candidates [6,7].

The vaccine was designed to preserve its fitness and immunogenicity. Individual genetic modifications contributed to the genetic stability and attenuation of the vaccine. In addition, their combination prevented detectable reversion to neurovirulence by reducing the capacity of the virus to acquire mutations that increase replication fitness in neuronal tissues [6].

In September 2015, wild poliovirus type 2 (WPV2) was certified as eradicated, leading to the withdrawal of OPV2 from routine immunization programs in April 2016. Following this, the use, study, and production of all type 2 polioviruses had to be completed under WHO Global Action Plan (GAP) III containment guidelines, which imposed restrictions such as limiting the handling and storing of polioviruses to only poliovirus-essential facilities [8]. Based on the GAP III restrictions and given that the nOPV2 candidates were not available for clinical evaluation before April 2016, additional measures were needed to study nOPV2 candidates. Anticipating these complexities, prior to the cessation of routine use of OPV2, several organizations from around the world partnered to rapidly plan and implement studies with Sabin OPVs, which would serve as historical control trials once nOPV2 preclinical development was complete. Primary study partners for phase I–II clinical trials included the University of Antwerp, Fighting Infectious Disease in Emerging Countries (FIDEC), Vaxtrials, CEVAXIN, International Centre for Diarrhoeal Disease Research, Bangladesh (icddr,b), and Assign, among others. Lab partners for sample evaluation included the US CDC, ViroClinics, and Dartmouth College [9]. An independent Data and Safety Monitoring Board (DSMB), with representation from global experts from different fields, monitored the safety findings of the EUL-informing studies. PATH sponsored the phase III clinical trial, partnering with Medical Research Council (MRC) Unit—The Gambia, and assisted with regulatory interactions, dossier preparation and coordination of the integrated product development efforts [10].

To comply with policy and regulatory requirements in the years following the global cessation of OPV and to avoid any accidental environmental release, the first-in-human study with nOPV2 was conducted in a fully contained, purpose-built facility named Poliopolis in Antwerp, Belgium, thereby ensuring that all biological samples potentially containing vaccine virus could be contained for subsequent decontamination [11]. This purpose-built unit was created through a collaborative effort across clinical and administrative staff at the University of Antwerp along with the local municipality and government as well as global stakeholders. The subsequent clinical trials with nOPV2 in Belgium and Panama that were conducted outside of contained settings closely mimicked the historical OPV2 control trials, as they were conducted at the same sites with the same investigators using the same protocol and procedures as the prior historic control studies with OPV2 [12,13,14]. This strategy was agreed upon with the relevant regulatory authorities prior to the initiation of the studies. Sites and countries were selected based on the feasibility of conducting the OPV studies in a timeframe relevant for developing a response tool for a Public Health Emergency of International Concern (PHEIC) and on assessment of a minimal risk of exposure of study volunteers to local poliovirus transmission, among other factors, to generate robust immunogenicity data. The regulatory authorities of the countries where clinical trials of nOPV2 were conducted played a key role in enabling the use of the study vaccines and related requirements for data collection, surveillance, and monitoring. Data from the phase I and phase II clinical studies in Belgium and Panama contributed to the selection of one of the two initially evaluated nOPV2 candidates and informed the eventual regulatory pathway [12,13,14].

## 3. Regulatory Pathway

In January 2020, the WHO published the EUL procedure, replacing the former Emergency Use Assessment and Listing (EUAL), which had been established in response to the Ebola outbreak of 2014–2016. The purpose of the EUL is to streamline access to unlicensed products through a risk-based assessment during public health emergencies [15]. The issuance of a WHO EUL is contingent on the vaccine first receiving an emergency use authorization (EUA) from the regulatory agency of record, usually the country in which the vaccine is produced. For nOPV2, which is manufactured by PT Bio Farma, the relevant regulatory agency is Badan POM (BPOM) in Indonesia.

In order to be eligible for an EUL, there are several criteria that must be met, including that the disease that the product is intended to treat or prevent is serious or life threatening, with the potential for outbreaks, epidemics, or pandemics, and that the existing products have not been able to eradicate or prevent the outbreaks. Given the sharp increase in cVDPV2 cases and outbreaks in 2019 and the risk of seeding additional outbreaks with continued OPV2 use in suboptimal quality response campaigns, nOPV2 was identified as a prime candidate for the EUL pathway. Further, without an EUL, nOPV2 would not be available for use until at least 2023–2024, following the availability of phase III clinical trial data for review of a marketing authorization and PQ dossier.

A dossier for an EUA and EUL was submitted to BPOM and the WHO, respectively, on 28 February 2020; an EUA was granted by BPOM on 12 November 2020, and an EUL was granted by the WHO on 13 November 2020, approximately three years from the time the first-in-human study was completed with nOPV2. nOPV2 was the first vaccine ever to receive EUL authorization.

Obtaining the EUL ahead of the completion of the phase III clinical study allowed nearly one billion doses of nOPV2 to be used over a span of nearly three years before the WHO prequalification of the vaccine. Following the completion of the phase III clinical trial at the MRC Unit—The Gambia, a dossier for marketing authorization and prequalification was submitted to BPOM and the WHO, respectively, in March 2023. nOPV2 was licensed in Indonesia on 23 December 2023^,^ and on 27 December 2023, nOPV2 was prequalified by the WHO [1].

## 4. Policy Framework

In February 2020, the WHO Executive Board issued a decision that called on the WHO Director-General (DG) to accelerate the assessment and rollout of nOPV2 [16]. This decision urged Member States to implement an expedited process for national approval of the importation and use of vaccines to respond to polio outbreaks, including nOPV2, on the basis of its anticipated EUL authorization, which included careful and rigorous analysis of available quality, safety and efficacy data. This decision paved the way for prioritization of nOPV2 review under the EUL pathway and was a powerful tool for advocating for countries to prepare proactively for nOPV2 use in case of need.

**Figure 2:**
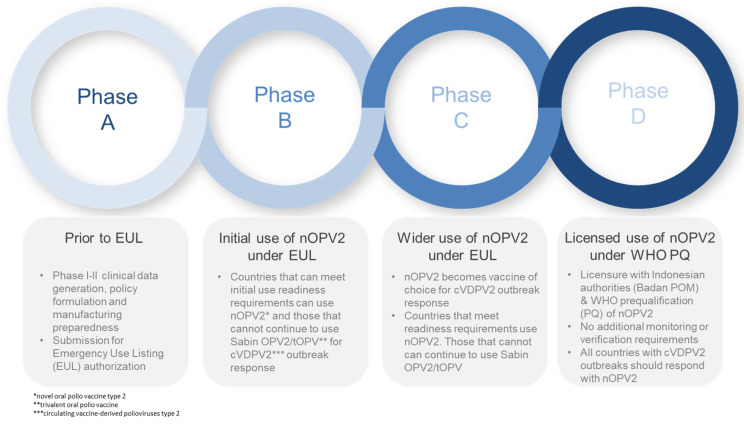
nOPV2 usage framework (endorsed by the Strategic Advisory Group of Experts to the WHO).

Engagement with the Strategic Advisory Group of Experts to the WHO (SAGE) provided the policy framework for nOPV2 development. Early on, as the EUL pathway was being advanced, the Global Polio Eradication Initiative (GPEI) sought SAGE’s endorsement of a phased rollout framework. The development of this framework (Figure 2) became a key feature of the risk mitigation approach under the EUL: the vaccine was first used under an ‘Initial Use’ phase, where countries had to meet a more stringent set of readiness verification requirements, followed by a ‘Wider Use’ phase, with fewer requirements.

While a range of additional data on genetic stability and effectiveness were collected and analysis was done to comply with EUL commitments, the determining factor for the recommendation to move beyond initial use was the absence of any concerning signals emerging from the analysis of safety data. SAGE made its recommendation on the basis of nOPV2 meeting safety milestones as assessed by a dedicated nOPV2 subcommittee established by the Global Advisory Committee on Vaccine Safety (GACVS).

More broadly, the independent review and guidance by SAGE were important for national acceptance and independent technical validation of the roll-out approach. Based on the available data and analysis, SAGE issued recommendations regarding nOPV2 use, which included endorsement for the clinical development of nOPV2, guidance on policies of nOPV2 use, and advice on phases of use under the EUL. SAGE also reviewed clinical and epidemiologic data and provided guidance on issues such as vaccine choice (nOPV2 vs. mOPV2 or tOPV), co-administration of nOPV2 with IPV, other parenterally administered childhood vaccines and interventions, and other OPV vaccines. Importantly, SAGE reiterated the importance of timely and high-quality campaign responses for vaccines to be most effective as outbreak response tools.

**Figure 3:**
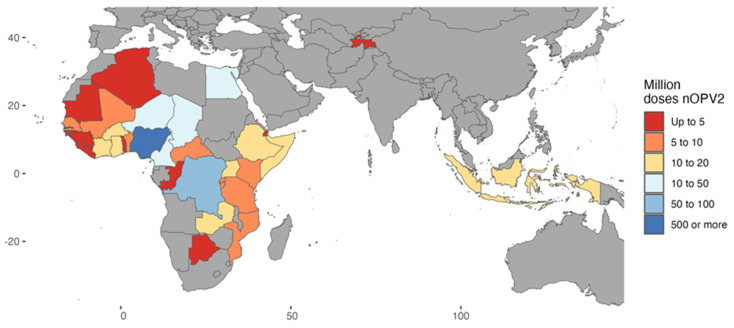
Scale of novel Oral Polio Vaccine Type 2 use for outbreak response under Emergency Use Listing authorization (between 1 March 2021 and 31 December 2023).

Under the EUL, governments of countries using nOPV2 chose to do so based on their own decision-making criteria; both national regulatory approval and documentation of a national decision (i.e., from a National Immunization Technical Advisory Group or the Ministry of Health) was required to be submitted as part of the readiness verification process. Regional and national decision making was supported by global guidance as well as the provision of regularly updated technical data and analysis. Thirty-five countries used nOPV2 during the EUL period (Figure 3). The majority of nOPV2 doses were used in Nigeria (approximately 500 million), followed by the Democratic Republic of the Congo (DRC) (approximately 68 million) [1].

## 5. Country Readiness for Deployment and De-Risking Supply

As with all OPV type 2 vaccines following the global switch in April 2016, nOPV2 is only available through a global stockpile under the oversight and release authority of the WHO DG. Given the monitoring requirements of the EUL, countries had to be verified for readiness to use nOPV2 prior to the DG’s release of the vaccine; those who did not meet these requirements could instead use OPV2, which was also available in the global stockpile. In the initial use phase, countries had to meet 25 readiness verification requirements across the seven areas referenced below, including meeting baseline thresholds for surveillance system performance. In its wider-use phase, there were no surveillance thresholds to meet, and the number of readiness requirements decreased to 16 across the same seven areas:

Coordination

Approvals (regulatory and national decision making)

Cold Chain and Vaccine Management (VM)

Surveillance

Safety

Advocacy, Communications, and Social Mobilization (ACSM)

Laboratories

### 5.1. Country Readiness

Countries were supported through the verification process jointly by WHO and UNICEF regional offices and nOPV2 regional (and, in some cases, country) focal points as well as a global readiness verification team. National Regulatory Authorities (NRAs) in countries where nOPV2 was used also contributed to the review of the vaccine and subsequent use of data under the EUL. The verification of requirements was conducted by GPEI subject matter experts and took place at both global and regional levels, depending on the requirement, and was overseen and coordinated at a global level. Countries were verified for nOPV2 use after all requirements were met. Verification was a one-time occurrence; once a country received verification, it could request nOPV2 during its EUL phase as needed in the future, without needing to go through the readiness verification process again.

Communications materials were developed to create a common understanding of nOPV2 development, processes, requirements, and rollout and provide technical content for those planning for and using the vaccine, with the intent of aligning technical and external communications. Multilingual guidance was developed to explain the process in general as well as to implement activities in each specific technical area. Materials were available for a range of stakeholders—from technical staff to ministers—in all key languages and were updated regularly. All information was available on a dedicated nOPV2 page within the GPEI website, including links to nOPV2-related SAGE and GACVS meeting summaries and recommendations, advocacy tools, and summaries of clinical development. GPEI partners held regular webinars and published an internal bulletin at a two-monthly interval, to share the latest findings for key GPEI staff. The WHO and UNICEF engaged at regional and country levels to support country decision making and planning for the use of nOPV2.

### 5.2. De-Risking Supply

One important element of preparation for nOPV2 use was early planning for supply of the vaccine. PT Bio Farma’s initial production was achieved through direct funding from the Gates Foundation for “at-risk” production of millions of doses. This forward-thinking action was critical in ensuring that the vaccine was available as soon as the EUL was granted, through a United Nations Children’s Fund (UNICEF) tender on behalf of GPEI for services to produce drug substances and finished products, including storing and managing a global stockpile. The manufacturer of nOPV2, PT Bio Farma, was responsible for producing and supplying all nOPV2 for the EUL period. To bolster the stability of the nOPV2 supply, a second manufacturer was subsequently identified, and as of early 2024, the process for review of the dossier for WHO prequalification of the second supplier was initiated. Despite the early planning and implementation of contingency measures around supply, there have been disruptions in global supplies of the vaccine due to a variety of reasons, which include supply chain challenges during the COVID-19 pandemic, occasional technical issues at the manufacturing facility, and increasing demand for the vaccine given the expansion of cVDPV2 outbreaks. A consistent and adequate supply of the vaccine remains a critical priority of the program to decisively interrupt ongoing cVDPV2 outbreaks.

## 6. Surveillance and Risk Mitigation

An EUL requires enhanced post-deployment monitoring of the vaccine. If quality or safety issues are identified during field use, or data gaps make monitoring results unreliable, the EUL procedure allows for the WHO to revoke the EUL recommendation. For nOPV2, these commitments included ongoing monitoring and assessment of the vaccine’s safety, genetic stability, and effectiveness, along with ensuring country systems were ready to detect and respond to any unanticipated findings. These commitments were outlined in the risk management plan (RMP), which was submitted to and accepted by Indonesian regulators and the WHO PQ as part of the EUL review process. While monitoring requirements vary by vaccine, unique factors to the nOPV2 EUL risk–benefit analysis included the fact that there was already a licensed polio vaccine (Sabin OPV2) to target type 2 outbreaks, that the vaccine would be used only for outbreak response (limited lead times to set up additional surveillance, i.e., sentinel surveillance sites), and that the countries most likely to use the vaccine were often those with complex field-level challenges (i.e., DRC, Nigeria, Yemen).

One of the key benefits for which nOPV2 was designed is its enhanced genetic stability compared with Sabin OPV2. Therefore, a key commitment under the EUL was to conduct intensive genetic stability analysis, including whole genome sequencing of isolates, which were reviewed on a regular basis, with findings summarized in initial monthly and subsequent quarterly reports. Samples evaluated in this pipeline came from the stool specimens of acute flaccid paralysis (AFP) cases and their contacts as well as environmental surveillance and also from cases of primary immunodeficiency involving long-term excretion of nOPV2. The whole genome sequencing was conducted by laboratories within GPEI’s Global Polio Laboratory Network (GPLN). A classification scheme was developed to categorize the results of the whole genome sequencing of isolates with respect to their relative level of concern regarding virulence [17].

Assays specific to the detection of nOPV2 were developed well in advance of nOPV2 use, and training was conducted at labs within the GPLN. To manage the number of poliovirus type 2 (PV2) isolates being sent to whole genome sequencing labs, a prioritization framework was put into place. Laboratory partners performed critical testing and analysis across a range of areas. The US CDC supported preclinical and clinical testing and analysis in support of nOPV2 studies. Once the EUL was in place, the US CDC, NIBSC, Institut Pasteur, and the Dutch National Institute for Public Health and the Environment (RIVM) performed whole genome sequencing on field isolates. The US CDC and NIBSC led the genetic characterization analysis and reporting effort, which was critical for compliance with EUL requirements and for understanding the new vaccine’s genetic characteristics in field use. These results were reviewed and discussed in a dedicated expert subgroup within the GPEI. Membership included PATH and a range of subject-matter experts in addition to GPEI partner agencies, who supported these efforts by providing context from the clinical development as well as recommendations for regulatory reporting.

Countries planning to use nOPV2 under an EUL were required to meet additional safety monitoring requirements and submit their data for consolidation at the global level, to ensure no concerning signals were missed. The GACVS Subcommittee on nOPV2 safety conducted periodic reviews of the safety data and analysis provided by countries using nOPV2 in order to provide an independent assessment of safety data emerging from countries during the EUL period. Through these reviews, the committee determined the presence or absence of any safety red flags and provided regular updates to both the full GACVS and SAGE, with summary reports published on the GPEI nOPV2 webpage.

**Figure 4:**
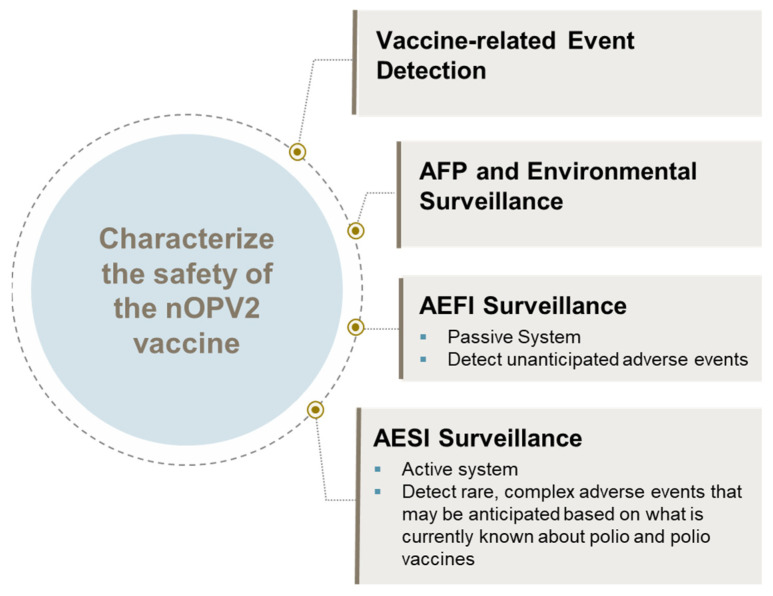
Safety monitoring framework.

Prior to the rollout of nOPV2, a number of possible conditions, designated as adverse events of special interest (AESIs), were identified, which constituted “safety signals” (Figure 4). To detect these, all countries using nOPV2 in the initial phases had to conduct active AESI surveillance as well as the standard adverse events following immunization (AEFI) surveillance. Over time, as the safety profile of nOPV2 became better characterized, it was noted by the GACVS that AFP surveillance formed the backbone of safety surveillance, as it captured conditions that were most likely to be related to AESIs from nOPV2 [18]. As such, in February 2022, the GACVS Subcommittee on nOPV2 Safety updated its guidance to note that active AESI surveillance [18] for nOPV2 safety was recommended but not required for countries without sufficient technical capacity and human resources to implement the active AESI protocol, thereby paving the way for additional countries to access the vaccine.

**Figure 5:**
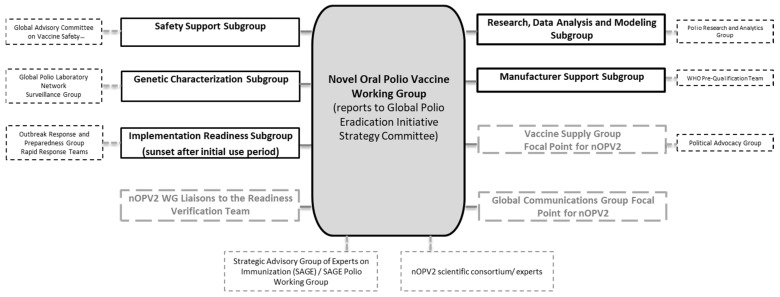
Novel Oral Polio Vaccine Working Group of Global Polio Eradication Initiative.

As the manufacturer of nOPV2, PT Bio Farma held accountability for meeting requirements for deployment under the EUL, and now under the PQ. Given the unique and complex nature of the commitments required under the EUL, the GPEI has supported PT Bio Farma in the funding, coordination, and implementation of these activities. In November 2019, the GPEI established a time-limited working group, the ‘nOPV Working Group’ (nOPV WG), to support PT Bio Farma in developing and implementing a monitoring plan to comply with requirements for the anticipated use of nOPV2 for outbreak response (Figure 5). The Working Group included representatives from all GPEI partners in its core group and established expert subgroups to guide key areas of work that would need to be implemented under the unique EUL conditions. Where technical groups already existed under the GPEI, the Working Group drew on the expertise of liaisons to these groups rather than establishing duplicative subgroups. The nOPV WG will sunset in 2024 based on the end of EUL use of the vaccine and the transition to being a WHO pre-qualified vaccine in December 2023. 

## 7. Assessment of Vaccine Effectiveness

The EUL for nOPV2 also required generating an evidence base regarding nOPV2 effectiveness in outbreak control. Evaluation of effectiveness in geographic regions eligible for outbreak response with nOPV2 presented significant challenges, given the lack of infrastructure in certain geographies to execute traditional vaccine effectiveness assessment studies. nOPV2 effectiveness in the field was assessed through multiple analytic methods: (1) interrupted time-series analysis, (2) time-Susceptible-Infectious-Recovered (tSIR) model-based analysis [19], and (3) a case–control study [20]. Because of their differing methodologies, each study required different types of data to assess different measures of nOPV2 effectiveness when used in outbreak response campaigns. Together, the results of these studies provided an understanding of the effectiveness of nOPV2 in the field during the EUL period.

The objective of nOPV2 development was to enhance the genetic stability of the existing vaccine while maintaining comparable effectiveness and safety to OPV2. Field and clinical data continue to support the enhanced genetic stability of nOPV2. Most nOPV2 isolates from AFP and environmental surveillance following supplementary immunization activities (SIAs) analyzed through whole genome sequencing indicate no or minimal changes in the genetic structure of nOPV2, while approximately 3% (as of September 2023) showed evidence of losing key stabilizing genetic modifications at domain V of the vaccine virus genome, compared with an expected 75% for mOPV2 [21]. A subset of these reverted strains led to 13 new cVDPV2 emergences, impacting 14 countries, as of February 2024. While any new emergence of cVDPV2 is a matter of serious concern, modelling assessment suggests that the risk of seeding cVDPV2 emergences following SIAs with nOPV2 is substantially reduced relative to SIAs with Sabin OPV2 [22]. Moreover, the primary mechanism of nOPV2 to revert to neurovirulent cVDPV2 strains, as demonstrated from the field data, is through a double recombination pathway that necessitates concurrent enterovirus infection in the gut, whereas with OPV2, single recombination events and point mutations can also lead to the generation of neurovirulent strains.

**Figure 6:**
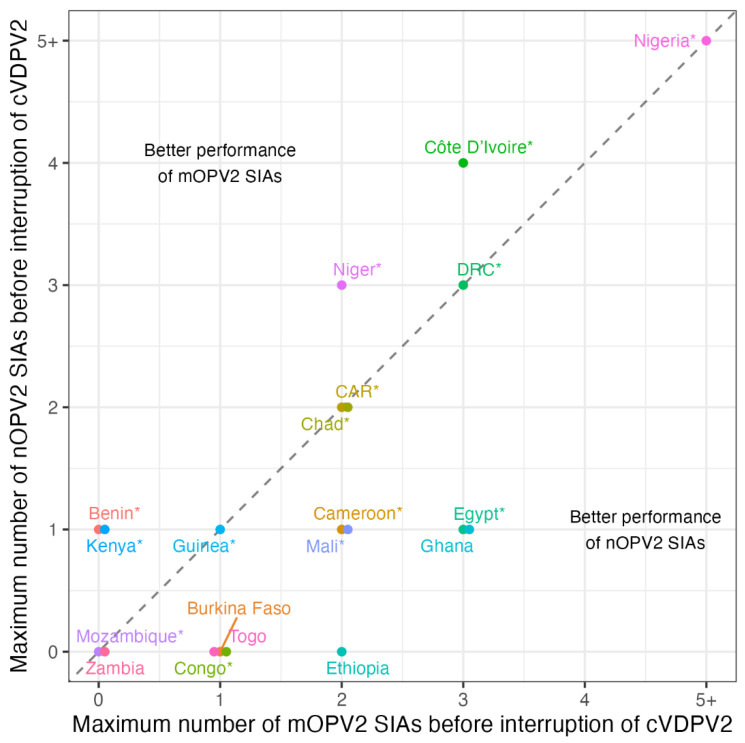
Maximum number of monovalent oral polio vaccine type 2 (mOPV2) or novel oral polio vaccine type 2 (nOPV2) supplementary immunization activities (SIAs) before interruption of circulating vaccine-derived polioviruses type 2 (cVDPV2) transmission in 19 countries that have used both vaccines (excluding Somalia, which also used trivalent oral polio vaccine (tOPV)). Interruption of transmission is defined as 180 days with no detection of cVDPV2 in poliomyelitis cases or environmental surveillance. Data were downloaded on 5 Feb 2024 from the Polio Information System [25]. Asterisks (*) indicate countries where the most recent detection is within 180 days of the most recent surveillance data, so interruption cannot yet be confirmed.

Data from field use and clinical trials support the hypothesis that nOPV2 would have similar immunogenicity and effectiveness to OPV2. Although findings of reduced immunogenicity of nOPV2 in young children when co-administered with bOPV indicated that nOPV2 may be less robust to competition with other enteroviruses in the gut [23], a study in the Gambia demonstrated excellent seroconversion in infants and young children (63% post-dose 1 in infants seronegative at baseline [n = 499], 65% in children [n = 66]), at levels comparable to those observed in an mOPV2 trial in a similar setting in Mozambique (68% in children seronegative at baseline [n = 75]) [10,24]. In addition, two in-depth studies comparing mOPV2 and nOPV2 effectiveness in Nigeria, which experienced a large cVDPV2 outbreak in 2021 and used the most nOPV2 of any country, found no significant difference between the two vaccines used in outbreak response campaigns [19,20]. Both nOPV2 and OPV2 campaigns were found to be highly effective in reducing transmission of cVDPV2. As noted, results from nOPV2 use in the field are consistent with the comparable individual immunogenicity of nOPV2 and mOPV2 found in clinical trials. More broadly, surveillance data suggest there is no substantial difference in the number of SIAs required to interrupt cVDPV2 transmission between nOPV2 and mOPV2 (Figure 6), with a few countries such as DRC and Nigeria requiring three or more SIAs to interrupt transmission irrespective of vaccine choice, and most countries achieving interruption with fewer than three SIAs.

## 8. Conclusions

**Figure 7:**
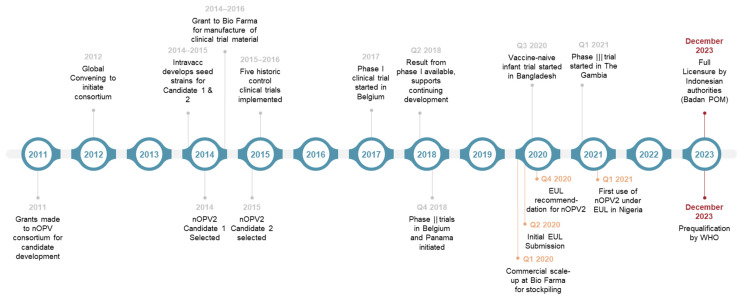
nOPV2: From idea generation to field use [adapted from 9].

The ultimate goal of the global polio eradication initiative is a world free from all forms of polioviruses. The development of nOPV2 is a significant advancement toward this audacious goal. Building on breakthroughs in genetic and vaccine science, and bringing together clinical, regulatory, and implementation partners, the nOPV2 development and rollout journey (Figure 7) is a shining example of a global collaboration to confront a public health emergency of international concern (PHEIC). Innovations in the clinical development of polio vaccines, such as conducting a phase I clinical trial in fully contained conditions, were matched with critical thinking on designing of the studies and development of novel assays to evaluate key study endpoints such as neurovirulence while maintaining the feasibility of the study population size. With countries from four out of six WHO regions joining the clinical development effort, it was a massive undertaking to generate data from different parts of the world with age groups ranging from adults to neonates, while effort was maintained to minimize interference on study endpoints from passive environmental exposure by avoiding trial sites in areas with ongoing polio outbreaks or with campaign use of OPVs [26]. Forward-looking decisions were taken on the manufacturing front to frontload production, with at-risk scaling up of production facilities at PT Bio Farma and with real-world innovations such as using a 50-dose vial to expand the availability of doses instead of the standard 20- or 10-dose vials. Consistent and planned communications with policymakers such as SAGE and independent expert groups such as GACVS ensured regular evaluation of the clinical and field data to inform and fine-tune use policies.

However, advances on the innovation front alone will be insufficient to achieve complete eradication. The critical importance of timely, high-quality outbreak response and sufficient vaccination coverage remain central to the strategy for global eradication of all forms of polioviruses. The urgency and ambition that underpinned nOPV2 development allowed for it to be the first vaccine approved under the EUL pathway with close to a billion doses used by countries in less than three years from its first use. In the coming years, in addition to strengthening essential immunization coverage of two or more doses of IPV, considerations for the use of nOPV2 in broader, preventative campaigns or select use in routine immunization schedules in specific settings may have to be evaluated if cVDPV2 circulation persists. Finally, it will really be the act of reaching every child in underserved communities with sufficient frequency and urgency to stop the transmission of vaccine-derived polioviruses from all corners of the earth. The overall development effort should also pave the way for accelerated and adaptive pathways for introducing critical response tools for other public health emergencies of international concern. Affordability of these tools for scaled-up production and accessibility to vulnerable populations for immunization response will continue to hold the key for a meaningful public health impact, much like what we have seen with the polio eradication effort and nOPV2 experience.

## Data Availability

All data used in the article available through GPEI resources or published materials.

## List of Acronyms and Abbreviations

AESIs	Adverse Events of Special Interest
AFP	Acute Flaccid Paralysis
bOPV	Bivalent Oral Polio Vaccine
BPOM	Badan POM (Indonesian Regulatory Authority)
cVDPV	Circulating Vaccine-derived Poliovirus
cVDPV2	Circulating Vaccine-derived Poliovirus type 2
DG	Director-General (of WHO)
DRC	Democratic Republic of the Congo
EUA	Emergency Use Authorization
EUAL	Emergency Use Assessment and Listing
EUL	Emergency Use Listing
GACVS	Global Advisory Committee on Vaccine Safety
GAP	Global Action Plan
GPEI	Global Polio Eradication Initiative
GPLN	Global Polio Laboratory Network
IPV	Inactivated Poliovirus Vaccine
mOPV2	Monovalent Oral Polio Vaccine type 2
MRC	Medical Research Council
NIBSC	National Institute for Biological Standards and Controls
nOPV	Novel Oral Polio Vaccine
nOPV2	Novel Oral Polio Vaccine type 2
nOPV WG	Novel Oral Polio Vaccine Working Group
OPV	Oral Polio Vaccine
OPV2	Oral Polio Vaccine type 2
PQ	Prequalification
PQT	Prequalification Team
SAGE	Strategic Advisory Group of Experts to the WHO
SIAs	Supplementary Immunization Activities
tOPV	Trivalent Oral Polio Vaccine
UNICEF	United Nations Children’s Fund
US CDC	US Centers for Disease Control and Prevention
VAPP	Vaccine-associated Paralytic Poliomyelitis
VDPV	Vaccine-derived Poliovirus
WHO	World Health Organization
WPV2	Wild Poliovirus type 2
